# Impacts and New Challenges with Highly Effective Modulator Therapies in Younger Children with Cystic Fibrosis

**DOI:** 10.3390/jcm14134625

**Published:** 2025-06-30

**Authors:** Kanchana Uyangoda, Charlotte Dawson, Nikesh Gudka, Rossa Brugha

**Affiliations:** Department of Respiratory Medicine and Cystic Fibrosis, Great Ormond Street Hospital, London WC1N 3JH, UK; kanchana.uyangoda@gosh.nhs.uk (K.U.); charlotte.dawson@gosh.nhs.uk (C.D.); nikesh.gudka@gosh.nhs.uk (N.G.)

**Keywords:** cystic fibrosis, highly effective modulator therapy, younger children

## Abstract

Highly effective modulator therapy (HEMT) has been available for adults and young adults aged 12 years and over with cystic fibrosis for approximately 5 years, with real-world evidence (RWE) emerging that confirms the significant impacts of these novel medications in older patient groups. As licensing has been extended to younger children (2 years and above in some jurisdictions), we summarize the clinical experience of these medications in pre-school and school-aged children and compare how changes in the objective markers of the disease can be elucidated in younger children. We also discuss the different incidences and severity of side effect profiles, the efforts to mediate these in younger children, and the particular challenges in introducing novel medications into pediatrics. We speculate on the use of HEMT in younger infants and its potential use in prenatal care.

## 1. Introduction

Cystic fibrosis (CF) is an autosomal recessive genetic disorder that affects approximately 100,000 individuals globally [1] and is caused by a mutation in the Cystic Fibrosis Transmembrane Conductance Regulator (*CFTR*) gene, located on the long arm of chromosome 7. This gene encodes the CFTR protein, an anion channel found on the apical surface of epithelial cells in sweat glands, airways, the gastrointestinal tract, the pancreas, and the vas deferens. CFTR plays a pivotal role in transporting chloride and bicarbonate across the epithelium and inhibiting the epithelial sodium channel (ENaC). Dysfunction in CFTR leads to reduced chloride secretion and increased sodium reabsorption. Consequently, secondary osmotic reabsorption of water occurs, causing dehydration of the epithelial fluids. Furthermore, the lack of bicarbonate results in reduced pH, which inhibits the activity of antimicrobial peptides in the airway surface liquid. These effects collectively contribute to the classic manifestation of CF, characterized by progressive lung disease, chronic infection, inflammation, and bronchiectasis. Additionally, exocrine pancreatic insufficiency causes malabsorption (impairing growth), and hepatobiliary disease leads to subsequent cirrhosis [1,2,3,4]. Since the initial discovery of the most prevalent CFTR variant (Phe508del) over three decades ago, more than 2000 different variants of CFTR mutations have been identified to date [5]. Among these, over 340 variants are classified as CF-causing variants and are present in the majority of CF patients worldwide [2]. The CFTR-causing variants are classified into six classes based on their structural function and their impact on protein synthesis, maturation, regulation, chloride conductance, and the level of normally functioning CFTR at the apical membrane. Class I variants disrupt protein synthesis, Class II variants affect protein folding (hence affecting the maturation), Class III variants alter channel regulation, Class IV variants influence chloride conductance, Class V variants reduce the quantity of normally functioning CFTR at the apical membrane, and Class VI variants diminish the stability of CFTR at the plasma membrane [6]. As the understanding of CF genetic variants has expanded, it has become evident that numerous variants exhibit the characteristics of multiple classes. The presence of Class I, II, or III variants results in a severe CF phenotype accompanied by pancreatic insufficiency, while Class IV, V, and VI variants with some residual function may be associated with milder disease [4,7].

CFTR modulators are small molecules that bind to specific sites on the CFTR protein and rectify CFTR dysfunction at the cellular level, thereby increasing the availability of the CFTR protein at the cell surface (correctors) or augmenting the gating function of the CFTR protein (potentiators). The development of CFTR modulators has significantly enhanced the clinical outcomes of patients with CF. CFTR modulators exhibit distinct modes of action depending on the targeted defects and include the following: potentiators (which prolong the open state of CFTR channels to enhance ion transport), correctors (which facilitate the correct folding and subsequent trafficking of the CFTR protein to the cell surface), amplifiers (which increase the CFTR protein production by improving the mRNA stability and translation), and stabilizers (which prevent CFTR degradation by maintaining protein stability at the plasma membrane) [4]. The development of CFTR modulators has progressed through several stages over the past decades. The first CFTR modulator was VX-770 (ivacaftor), a potentiator approved in 2012 that benefited patients with gating variants like G551D, including younger children [8,9,10]. However, for patients with the most common CFTR variant (Phe508del), ivacaftor alone was not effective. To address this, VX-809 (lumacaftor), a corrector, was combined with ivacaftor and approved as Orkambi in 2015 for Phe508del homozygous patients, though with limited efficacy in comparison to the group of patients who had benefitted from ivacaftor alone [11,12]. Lumicaftor was followed by VX-661 (tezacaftor), a refined corrector with improved pharmacokinetics and fewer side effects; in combination with ivacaftor, this was approved in 2018 for Phe508del homozygous and Phe508del heterozygous patients with a limited list of specific residual function variants, in patients aged greater than 12 years [13]. A significant advancement came with the development of VX-445 (elexacaftor), a “next-generation” corrector, combined with tezacaftor and ivacaftor (ETI) and marketed as Trikafta^®^/Kaftrio^®^, which was approved in 2019 [14]. ETI offers clinical benefits for patients with at least one Phe508del mutation, with emerging evidence that ETI may also improve lung function and reduce exacerbations in patients who do not carry a single Phe508del variant on the basis of rarer genotypes [15,16]. The progression from single-agent potentiators to highly effective triple-combination therapies has revolutionized CF treatment, extending improved outcomes to a broader range of patients. The newest addition to the triple-combination modulator therapy, Vanzacaftor/tezacaftor/deutivacaftor (Alyftrek), was approved by the FDA in December 2024 for CF patients aged 6 years and older who have a mutation that is eligible for ETI (Trikafta) or one of 31 rare mutations. Alyftrek only needs to be taken once a day, making the dosing regimen more convenient as well as offering another option for patients who cannot tolerate ETI [17].

## 2. Evidence for Highly Effective Modulator Therapy in Patients over 12 Years of Age with CF

Clinical trials conducted in adolescents and adults demonstrate the efficacy and safety of HEMT, with substantial evidence supporting its use in older age groups in a number of key studies. A multicenter phase 3, randomized double-blind placebo-controlled trial evaluated the efficacy and safety of elexacaftor–tezacaftor–ivacaftor (ETI) in 403 patients aged 12 years and older with cystic fibrosis and Phe508del–minimal function genotypes. Patients who had an FEV1 of 40–90% and who had stable disease during a 28-day screening period were randomly assigned to receive ETI or a placebo for a 24-week period. Over 24 weeks, ETI significantly improved lung function (the percent predicted FEV1 increased by 13.8 points at 4 weeks and 14.3 points through 24 weeks), reduced pulmonary exacerbations (63% lower rate versus placebo), decreased sweat chloride by 42 mmol/L, increased CFQ-R respiratory domain scores by 20 points, and improved BMI values by 1.04 kg/m^2^ [18]. An additional phase 3, multicenter (96 sites), double-blind, parallel-group, randomized, active-controlled trial evaluated the efficacy and safety of ETI in patients with cystic fibrosis who had Phe508del-gating or Phe508del-residual function genotypes. A total of 271 patients had entered a 4-week run-in period with ivacaftor or tezacaftor–ivacaftor. Following this run-in period, 258 patients were randomized to receive ETI (*n* = 132) or to continue their previous regimen (*n* = 126) for 8 weeks. The treatment groups were well matched at the baseline. ETI resulted in greater improvements in percent predicted FEV1 (ppFEV1), sweat chloride levels, and quality of life compared to the control group [14]. These findings were further supported by the PROMISE study, a prospective, observational study in 487 U.S. patients aged 12 and older with at least one F508del allele, which demonstrated the real-world effectiveness of ETI [19]. Over 6 months, ETI significantly improved FEV1, reduced sweat chloride levels, and enhanced respiratory quality of life, while also showing positive effects on BMI values. A real-world study utilizing registry data from 2645 individuals with cystic fibrosis from 67 centers in Germany showed similarly significant clinical outcomes with ETI treatment over a 12-month period [20].

## 3. Effectiveness of HEMT in Younger Children

HEMT has emerged as a transformative therapy for children under 12 years of age with cystic fibrosis, demonstrating improvements in measurements of lung function, respiratory symptoms, and quality of life. A phase 3b, randomized, double-blind, placebo-controlled multicenter study evaluated the efficacy and safety of ETI in 121 children aged 6 to 11 years with cystic fibrosis who were heterozygous for F508del and had a minimal function mutation and LCI more than or equal to 7.5 [21]. The patients were randomized (1:1) to receive ETI (*n* = 60) or a placebo (*n* = 61) over 24 weeks. ETI significantly improved clinical outcomes over 24 weeks and reduced the lung clearance index (a measure of ventilation homogeneity, where higher values imply increased gas trapping secondary to CF lung disease [22]) by 2.29 units compared to 0.02 units in the placebo group, and it increased ppFEV1 by 9.5% versus a 1.5% decrease in the placebo group. Sweat chloride levels decreased by 52.1 mmol/L with ETI compared to 0.9 mmol/L in the placebo group, with 81.7% of the ETI-treated children achieving sweat chloride levels below 60 mmol/L. Respiratory symptoms, measured by CFQ-R respiratory domain scores, improved by 5.9 points with ETI versus 0.5 points in the placebo group [21]. Another phase 3, open-label, multicenter study evaluated the safety and efficacy of ETI in 66 children aged 6 to 11 years with cystic fibrosis and at least one F508del allele over 24 weeks [23]. The genotype and the basis of the diagnosis of CF had been confirmed at the screening, and all patients had stopped or remained off CFTR modulators for more than 28 days before the first visit. This study demonstrated significant improvements in lung function ppFEV1 (increased by 10.2%), lung clearance index (decreased by 1.71), sweat chloride concentrations (decreased by 60.9 mmol/L), and CFQ-R respiratory domain scores (increased by 7 points). It also showed improvements in body mass index (BMI) values and BMI-for-age z-scores over the 24-week period [23]. These findings were confirmed in a phase 3, open-label extension study, which evaluated the long-term safety and efficacy of ETI in 64 children aged 6 years and older with CF and at least one F508del allele over 96 weeks. The patients using ETI maintained significant improvements in lung function, sweat chloride levels, respiratory symptoms, and growth parameters, with a favorable safety profile [24]. The long-term safety and efficacy were further established by the second part of this extension study, in which the children who were more than 6 years old and on ETI treatment were followed up for 192 weeks. The results showed a similar favorable safety profile with no new adverse events, along with a clinically meaningful improvement in pulmonary function tests, CFTR function, and nutritional status on long-term follow-up. A retrospective, real-world study assessed the intermediate-term effects of ETI in 46 children aged 6 to 17 years who had started ETI between August 2020 and October 2022 [25]. Over 6 months, the use of ETI resulted in significant improvements in lung function (ppFEV1 increased by 11.4 percentage points), sweat chloride concentrations (decreased by 59.3 mmol/L), and BMI-for-age z-scores (increased by 0.31 points at 3 months, sustained through 6 months) [25].

Building on the encouraging results observed in studies involving children aged 6 years and older, research efforts were subsequently extended to evaluate the efficacy of ETI in younger children, aged 2 to 5 years, aiming to assess its potential benefits in this earlier stage of the disease. A phase 3, open-label, two-part, multicenter international study assessed the safety, pharmacokinetics, pharmacodynamics, and efficacy of ETI. The first part was conducted in 18 children in seven sites in the U.S. to assess the safety and tolerability of ETI over a 15-day period and to determine the dose to be used in part B of the study. Part B was conducted in 22 international sites over 24 weeks in 75 children aged 2 to 5 years with at least one F508del allele [26]. The study demonstrated significant improvements in sweat chloride concentrations and lung clearance index (LCI2.5). Growth parameters remained stable, and the safety profile was consistent with findings in older children and adults. These results support the early use of ETI in children as young as 2 years old to improve the clinical outcomes and potentially delay the disease progression [26].

Until recently, elexacaftor–tezacaftor–ivacaftor (ETI) was approved only for individuals with cystic fibrosis (CF) who carried at least one copy of the Phe508del mutation, the most common CFTR mutation in Caucasian populations. However, this mutation may be less prevalent in non-Caucasian populations, restricting their eligibility for ETI treatment. Recent studies evaluating the effectiveness of ETI in patients with non-Phe508del mutations have shown promising results [15,16]. As a result of this, since 2023, ETI has been approved for the treatment of CF, including in younger children (aged 2 years and above) and for use with a broad range of non-Phe508del CFTR variants. Of note, ivacaftor monotherapy for responsive CFTR variants is approved for infants aged 4 months and above in the UK, and the FDA has approved its use in children as young as 1 month.

## 4. Extrapulmonary Effects of HEMT in Younger Children

In addition to its substantial impact on lung function and sweat chloride levels, the utilization of HEMT has demonstrated remarkable improvement in the weight and body mass index (BMI) of younger children. It has also been shown to increase both the fat mass and fat-free mass in the pediatric population [27]. Fat-soluble vitamin levels, particularly vitamin A levels, are increased following the commencement of highly effective modulator therapy in children and adults [28,29]. The ETI-induced reduction of pulmonary gut inflammation and improved uptake of fat-soluble micro-nutrients secondary to enhanced pancreatic function are proposed mechanisms [30]. Young children with gating mutations who are treated with ivacaftor exhibit improved exocrine pancreatic function, as evidenced by elevated fecal elastase and reduced immunoreactive trypsinogen levels [31,32]. The use of HEMT has also demonstrated notable benefits in managing chronic rhinosinusitis in children with cystic fibrosis by reducing sinus inflammation and improving mucociliary clearance [33,34,35].

## 5. Once-Daily Modulator Therapy in Children

The newest combination highly effective modulator therapy (HEMT) is a once-daily next-generation combination therapy, vanzacaftor-tezacaftor-deutivacaftor, which is approved for use in children aged 6 years and older. In a trial conducted in patients aged 12 years and older, vanzacaftor-tezacaftor-deutivacaftor was non-inferior to ETI with respect to ppFEV1 while demonstrating a superior restoration of CFTR function, evidenced by a significant reduction in sweat chloride levels compared to ETI [36]. The subsequent trial in children aged 6–11 years showed that vanzacaftor-tezacaftor-deutivacaftor was safe and well tolerated in this younger cohort, alongside similar findings with respect to ppFEV1 and sweat chloride levels [17].

## 6. Adverse Effects of HEMT in Children

While HEMT is generally well tolerated in younger children, a range of adverse effects (many similar to those reported in older children and adults) has been documented. Most adverse events are mild to moderate in severity and frequently overlap with common manifestations of CF or typical pediatric infections [21]. Commonly reported adverse effects in younger children receiving HEMT include cough, headache, rhinorrhea, nasal congestion, pyrexia, and upper respiratory tract infections [26].

Elevated transaminase levels, particularly alanine aminotransferase (ALT) and aspartate aminotransferase (AST), are a recognized adverse event associated with HEMT in younger children, and the careful monitoring of liver function is essential following the initiation of the therapy. A phase 3 clinical trial in children aged 6–11 years reported elevated transaminase levels exceeding three times the upper limit of normal (ULN) in 10.6% of the participants, with 1.5% of the participants exceeding five times the ULN [23]. An extension study demonstrated a reduced incidence of elevated transaminases after 96 weeks, adjusted for exposure, compared to the parent study [24]. Similarly, in phase 3 trials conducted in children aged 2–5 years who were receiving elexacaftor-tezacaftor-ivacaftor (ETI), elevated transaminase levels were observed in six children, with two cases exceeding five times the ULN and one case exceeding eight times the ULN [26]. Ivacaftor safety trials in children aged 2–5 years and under 1 year showed comparable trends, with one case requiring treatment discontinuation due to transaminase levels exceeding eight times the ULN, while other cases required a temporary treatment interruption followed by a successful reinitiation at a lower dose after the transaminase levels had normalized [10,37].

The development of a rash following HEMT is another commonly reported adverse effect in younger children. The phase 3 clinical trials conducted in 2–5-year-old children on ETI showed a 20% incidence of a rash event [26]. Similar patterns were observed in children aged 6–11 years on ETI treatment, with descriptions ranging from erythematous and maculopapular reactions to papular responses, skin exfoliation, and urticaria [21]. Most of the rashes were mild to moderate in severity. One child discontinued treatment due to a rash after the first dose, while two children required a temporary treatment interruption, followed by a successful reinitiation.

Cataract formation is a well-recognized adverse effect of ivacaftor, identified in preclinical studies and multiple clinical trials, prompting the U.S. Food and Drug Administration (FDA) to recommend ophthalmologic evaluations before and after HEMT initiation in younger children [31]. Phase 3 clinical trials conducted in children aged 2–5 years [10] and below 1 year [37] reported no new-onset cataracts or lens opacities, except for a single case of lens opacity identified during the extension study [38]. Similarly, phase 3 ETI trials in children aged 2–5 years demonstrated no new or unexpected ophthalmologic events. However, a recent case series reported bilateral cataracts in three newborns exposed to ETI in utero and during breastfeeding, with no other identifiable risk factors for congenital cataracts [39]. These findings emphasize the importance of active surveillance in newborns born to mothers receiving ETI and highlight the need for the continued monitoring of infants on HEMT to detect potential ophthalmologic adverse events.

Emerging evidence suggests that HEMT may be associated with behavioral and mental health effects in younger children. A recent real-world study in children aged 6–11 years receiving ETI reported adverse effects, including constipation and behavioral and mental health concerns, which required dose adjustments [40]. Similarly, a French real-world study involving 197 preschool children across 34 CF centers documented behavioral and sleep disturbances in 47% of children within one month of ETI initiation [41]. Reported behavioral changes included symptoms resembling attention deficit hyperactivity disorder (ADHD), irritability, aggressive and oppositional behavior, and mood disturbances, with one case of suicidal ideation. Sleep-related disturbances, such as difficulty falling asleep, frequent night awakenings, and nightmares, were also observed, persisting for up to 3 months post-initiation [41]. These findings highlight the need for the careful monitoring of behavioral and sleep-related effects in younger children receiving HEMT.

In contrast to studies in adults, to the best of our knowledge, clinical trials in younger children have not reported significant elevations in creatine kinase exceeding five times the ULN or adverse events related to hypertension. Taking an overall perspective, the data from a large multicenter retrospective study (*n* = 608) indicates that approximately 18% of children have an adverse reaction to ETI, the majority of these reactions are temporary, and only 1% of patients need to cease treatment [42].

## 7. Real-World Approaches to Complications of HEMT

Drawing from our experience, we wish to present anecdotal evidence on the use of highly effective modulator therapies in a younger patient cohort (<12 years) treated at the Great Ormond Street Hospital CF service. The CF clinic comprises 198 children with CF (CwCF), of which 32 meet the eligibility criteria for HEMT based on age, genetic CF variant, and a confirmed diagnosis of CF. All CwCF are reviewed in the CF clinic every 2–3 months as per the current UK Standards of Care for the Management of CF (Available on https://www.cysticfibrosis.org.uk/sites/default/files/2024-08/Standards%20for%20the%20clinical%20care%20of%20children%20and%20adults%20with%20cystic%20fibrosis%20in%20the%20UK%202024.pdf accessed on 9 April 2025). Following the introduction of HEMT, parents of CwCF receive a telephone follow-up 24 h post-D1 to check for any issues with the dose administration or initial side effects and to confirm the follow-up plans. The patients’ growth, age-appropriate spirometry, and liver transaminases are measured at 4–6 weeks, 3 months, 6 months, 9 months, and 12 months in the CF clinic for the first-year post-HEMT introduction.

The adverse effects observed in our clinic are largely consistent with the currently reported evidence, and HEMT is generally well tolerated by our patients. The majority of younger children experience elevated transaminases (ALT or AST), with only a few maintaining normal liver function. However, none of the patients met the criteria for discontinuing treatment. A small number have required dose adjustments following a temporary pause in medication. For those with altered liver enzymes, the treatment is paused for one month, after which a modified dose is introduced once liver function tests are normalized or near-normalized. HEMT is then gradually increased, with transaminases monitored every 2 weeks. Compared to adults, the frequent blood testing for liver enzyme monitoring presents a significant challenge in managing younger children. Constipation is another observed adverse effect, likely associated with improved fat absorption, which has necessitated a reduction in the pancreatic enzyme replacement therapy (PERT) dosage in some patients. Additionally, patients may require dietary management due to excessive weight gain, secondary to an increased appetite and enhanced nutrient absorption. As reported in the literature, elevated vitamin A levels have been detected in patients aged 2–5 years, as well as those over 6 years, during routine annual assessments, leading to dose adjustments or new preparations of vitamin supplements for many of our younger patients. Elevated levels of vitamins D and E have not been observed. An increase in wet cough was commonly reported following the initiation of HEMT, though this typically resolved over the first few weeks.

A small number of patients have experienced issues related to sleep, behavior, and mental health. Their parents have reported difficulties such as trouble falling asleep, frequent awakenings, and nightmares. As a first-line intervention, a non-pharmacological approach, including sleep hygiene advice, is recommended. Some patients take their evening dose of ivacaftor earlier, while a few omit the evening dose. Behavioral issues, including emotional and angry outbursts, are predominantly reported in the youngest age group (2–5 years), whereas mental health concerns, such as a low mood, were observed in patients aged 6–11 years. These patients are referred to a CF psychologist for counseling sessions, though no dose adjustments to HEMT have been necessary. Notably, these effects did not become evident within the first 6–9 months of the HEMT initiation and were not reported by schools or nurseries when asked. It remains unclear whether these adverse effects are attributable to HEMT, developmental stages in childhood, or wider factors. Further research is needed to evaluate the potential link between these effects and the usage of HEMT in younger children, as this presents a significant diagnostic challenge.

## 8. Future Directions

The use of HEMT has now expanded significantly, with trials extended into the 1–2-year-old age group (ClinicalTrials.gov ID NCT05882357). There is emerging evidence suggesting its potential benefits in utero. Notably, fetuses have demonstrated clinical improvements, including the clearance of meconium ileus, when mothers were treated with ETI during the third trimester.

A real-world study examining maternal–CF fetal dyads, where fetuses were diagnosed with CF and had at least one responsive variant, showed that the maternal use of ETI or ivacaftor in the third trimester effectively cleared meconium ileus within 14 days, with no concerns regarding fetal development and acceptable neonatal tolerance of highly effective modulator therapy (HEMT), further supporting its potential role in prenatal and early-life CF management [43].

The impact of HEMT on CFTR function early in life poses unique questions to the assumed, established pathophysiology of CF in infancy and early childhood. Early lung inflammation and infection are likely to be reduced, and the use of prophylactic antibiotics in early childhood is challenged by the use of HEMT in this group; trials of antibiotic prophylaxis (for example, CF-START [ISRCTN18130649]) will need to be interpreted in this context. Changes in pancreatic function are the major challenge, with a number of groups presenting conference papers in the past 12 months showing changes in pancreatic function in young children on HEMT and protocols emerging for the cessation of pancreatic enzyme replacement therapy; it would be reasonable to speculate that there may also be a downstream effect on the endocrine pancreas function and peripheral insulin resistance later in life such that the rates of CF diabetes diagnoses may fall, or the onset may be postponed into later adult life. Sinus and gut issues may become less common. Finally, if HEMT is to be started following a prenatal diagnosis, there may be an impact on the formation of the vas deferens, with a subsequent improvement in male fertility. The community of CF clinical teams will need to work in combination to agree on the processes and protocols to address the monitoring of these multi-system impacts on the use of HEMT in children growing up with a diagnosis of CF.

## 9. Conclusions

HEMT is now an established therapy in many countries. HEMT use is expanding into patients without the Phe508del variant, as well as into younger age groups, and parents and professionals are advocating for its use in antenatal scenarios. Countries with mature CF registries are well placed to conduct the well-designed phase 4 monitoring of these medications and their next iterations. There is a significant knowledge gap in the cystic fibrosis community with respect to the approaches to side effects with this class of medications, and while a “one size fits all” approach to raised transaminases or behavior changes is unlikely to be practical, future clinical trial designs could incorporate stepwise protocols for pauses and a reduced-dose reintroduction of these novel molecules.

## Abbreviations

The following abbreviations are used in this manuscript:

CF	Cystic Fibrosis
HEMT	Highly Effective Modulator Therapy
CFTR	Cystic Fibrosis Transmembrane Conductance Regulator
ETI	Elecacaftor-Tezacaftor- Ivacaftor
PERT	Pancreatic Enzyme Replacement Therapy
ULN	Upper limit of Normal
ALT	Alanine amino Transferase
AST	Aspartate Amino Transferase
BMI	Body Mass Index
